# Impact of socioeconomic status on chronic control and complications of type 1 diabetes mellitus in users of glucose flash systems: a follow-up study

**DOI:** 10.1186/s12916-024-03254-w

**Published:** 2024-01-25

**Authors:** Fernando Sebastian-Valles, Julia Martínez-Alfonso, Jose Alfonso Arranz Martin, Jessica Jiménez-Díaz, Iñigo Hernando Alday, Victor Navas-Moreno, Teresa Armenta Joya, Maria del Mar Fandiño García, Gisela Liz Román Gómez, Jon Garai Hierro, Luis Eduardo Lander Lobariñas, Purificación Martínez de Icaya, Miguel Antonio Sampedro-Nuñez, Vicente Martínez-Vizcaíno, Mónica Marazuela

**Affiliations:** 1 Department of Endocrinology and Nutrition, Hospital Universitario de La Princesa, Instituto de Investigación Sanitaria de La Princesa, Universidad Autónoma de Madrid, 28006 Madrid, Spain; 2 Department of Family and Community Medicine, Centro de Salud Daroca, 28006 Madrid, Spain; 3 Department of Endocrinology and Nutrition, Hospital Universitario Severo Ochoa, Leganés, 28194 Madrid, Spain; 4 Department of Endocrinology and Nutrition, Hospital Universitario Basurto, 48013 Bilbao, Spain; 5 Health and Social Care Research Center, Universidad de Castilla-La Mancha, 16071 Cuenca, Spain; 6 Facultad de Ciencias de La Salud, Universidad Autónoma de Chile, Talca, Chile

**Keywords:** Continuous glucose monitoring, Socioeconomic status, Diabetes technology, Health inequalities, Socioeconomic deprivation, T1D

## Abstract

**Background:**

This study investigates the association between socioeconomic status (SES) and glycemic control in individuals with type 1 diabetes (T1D) using flash glucose monitoring (FGM) devices within a public health system where these technologies are freely available and utilized according to recommended guidelines.

**Methods:**

A follow-up study of 1060 adults (mean age 47.4 ± 15.0 years, 49.0% women) with T1D, receiving care at three Spanish university hospitals that regularly employ the FGM system. SES was assessed using the Spanish Deprivation Index and the average annual net income per person. Glycemic data were collected over a 14-day follow-up period, including baseline glycated hemoglobin (HbA1c) levels prior to sensor placement, the last available HbA1c levels, and FGM-derived glucose metrics. Individuals with sensor usage time < 70% were excluded. Chronic micro and macrovascular complications related to diabetes were documented. Regression models, adjusted for clinical variables, were employed to determine the impact of SES on optimal sensor control (defined as time in range (TIR) ≥ 70% with time below range < 4%) and disease complications.

**Results:**

The average follow-up was of 2 years. The mean TIR and the percentage of individuals with optimal control were higher in individuals in the highest SES quartile (64.9% ± 17.8% and 27.9%, respectively) compared to those in the lowest SES quartile (57.8 ± 17.4% and 12.1%) (*p* < 0.001). Regression models showed a higher risk of suboptimal control (OR 2.27, *p* < 0.001) and ischemic heart disease and/or stroke (OR 3.59, *p* = 0.005) in the lowest SES quartile. No association was observed between SES and the risk of diabetic nephropathy and retinopathy. FGM system improved HbA1c levels across all SES quartiles. Although individuals in the highest SES quartile still achieved a significantly lower value at the end of the follow-up 55 mmol/mol (7.2%) compared to those in the lowest SES quartile 60 mmol/mol (7.6%) (*p* < 0.001), the significant disparities in this parameter between the various SES groups were significantly reduced after FGM technology use.

**Conclusions:**

Socioeconomic status plays a significant role in glycemic control and complications in individuals with T1D, extending beyond access to technology and its proper utilization. The free utilization of FGM technology helps alleviate the impact of social inequalities on glycemic control.

**Supplementary Information:**

The online version contains supplementary material available at 10.1186/s12916-024-03254-w.

## Background

Since the publication of the results of The Diabetes Control and Complications Trial [1] the evidence supporting that tight glycemic control can buffer the adverse effects of hyperglycemia in patients with type 1 diabetes (T1D) has continued to grow [1]. It has also been demonstrated that in adults with T1D, continuous glucose monitoring (CGM) systems improve glycemic control in all age groups and education levels [2]. Concurrently, the metrics offered by CGM devices have attained parity with, and in some cases, surpassed HbA1c as measures of glycemic regulation [3].

The role of social determinants of health [4] in the chronic control of diabetes, as measured by glycated hemoglobin [5, 6], in the development of complications [7, 8], and even in mortality [9, 10], has been extensively addressed to date. The advantages and widespread use of CGM systems in T1D patients suggest that disparities could be reduced [11] by the free access to these technologies [12]. However, according to Hart’s inverse care law [13], self-management of disease interventions without an adaptation and targeting of the barriers that may arise, may not only fail to mitigate the effect of social inequalities on health but may even aggravate it [14].

In many developed countries, including Spain, adults with T1D are provided with a reimbursed Flash CGM system (FGM) by the national health system. This initiative aims to improve diabetes management and reduce health disparities. Therefore, the objective of this study was to assess the relationship between the deprivation index, used as a measure of SES, and glycemic control in individuals with T1D who utilize FGM systems. This study was conducted in a setting where these systems are readily available and used in accordance with recommended guidelines. Additionally, the study aimed to compare these findings with those of the control group before the implementation of FGM systems.

## Methods

This follow-up study included 1060 individuals attending three Spanish hospitals (Hospital Universitario Basurto, Hospital Universitario de la Princesa, and Hospital Universitario Severo Ochoa), located in three different geographical areas. All the participants were regular users of FGM (FreeStyle Libre ®, Abbott), with mean time since first use 2.3 ± 1.4 years. Glucose metrics were collected from cloud downloads on the Libreview platform over a 14-day period in October 2022. Furthermore, HbA1c levels were assessed 1 month prior to initiating sensor usage, and the HbA1c value closest to the extraction of glucose metrics from the FGM platform was also obtained.

Inclusion criteria were diagnosis of T1D and regular use of FGM. Exclusion criteria were patients with a diagnosis of type 2, MODY, or other types of diabetes, those with a usage time < 70%, and those who did not have a download of sensor data in the 30 days before data collection (initial cohort 2115 individuals). This study followed the “Strengthening the Reporting of Observational Studies in Epidemiology (STROBE)” guidelines [15]. The study was approved by the Research Ethics Committee of Hospital de La Princesa, Madrid (Study number: 5084–01/2023). The research was conducted according to the Declaration of Helsinki.

### Procedures

Prior to the start of the FreeStyle monitor, all patients received a training session on the use of the monitor according to international recommendations [16]. The system consists of a glucose oxidase–based electrochemical sensor placed subcutaneously that is replaced every 14 days, along with a receiver to which interstitial glucose measurements are sent wirelessly and stored in the cloud using the Libreview platform. All patients were provided with written instructions on how to use the data provided by FGM to make real-time adjustments of insulin doses and on the use of Libreview cloud to retrospectively review the glucose data to adjust future insulin doses. All patients were instructed to adjust their insulin dosages and hypoglycemia treatment in accordance with their glucose profiles and trends.

### Data collection

Glucose metrics were retrieved from the Libreview platform using the FreeStyle 2 device (FreeStyle Libre 2®, Abbott) at 14-day intervals. The following variables were collected: time in range (TIR), time below and above range ([TBR], glycemia < 70 mg/dL and time above range[TAR], glycemia > 180 mg/dL, respectively), number of daily readings, sensor usage, coefficient of variation (CV) and standard deviation (SD). Glycemic control was considered optimal when participants had a TIR > 70% and a TBR < 4%, as recommended [16].

In addition, sociodemographic and clinical data, as well as laboratory tests and pharmacologic medication for T1D, were collected from electronic health records, including sex, age, diabetes mellitus duration, type of diabetes, body mass index (BMI), smoking behavior, use of continuous subcutaneous insulin infusion (CSII), baseline HbA1c, last available HbA1c, FGM usage time, age at disease onset, insulin dose, nephropathy, and retinopathy. Both complications were classified following international standard [17–19]. We also considered a “non–fatal cardiovascular event” as a composite variable combining ischemic heart disease and non-fatal ischemic stroke. Glycated hemoglobin was routinely determined using liquid chromatography (ADAMS A1c HA8180 V ARKRAY®).

### Socioeconomic status (SES): deprivation index and mean net annual income per person

SES in Spain was assessed using the deprivation index for the entire Spanish territory based on census section, 2021 [20]. It combines information about the following variables for each census tract: manual working population, casual wage-earning population, unemployment, people aged 16 and over and 16 to 29 years with insufficient education, and main households without Internet access. This index was categorized into quartiles (low, medium–low, medium–high, and high-deprived neighborhoods, corresponding to the first, second, third, and fourth quartiles, respectively). Throughout the text, the SES will be represented by the deprivation index.

Additionally, as a sensitivity analysis, we also used the mean annual net income per person for each census tract (Additional file 1: Fig. S1), which is periodically published by the National Institute of Statistics (2019); thus, this variable was used in a similar way to the deprivation Index (National Institute of Statistics of Spain. (2020). Atlas de Distribución de Renta de los Hogares 2020. Retrieved from https://www.ine.es/componentes_inebase/ADRH_total_nacional.htm Accessed 7 August 2023.

### Statistical analysis

After checking for the plausibility of the outliers, data fit to the normal distribution was examined by statistical (Kolmogorov–Smirnov test) and graphical (normal probability plot) procedures. Those variables with extreme values, whose authenticity was verified, were trimmed using the 99th and 1st percentiles of the distribution. Continuous variables were presented as mean and standard deviation (SD) and categorical variables as numbers and percentages of samples.

Pearson correlation coefficients were estimated to examine the relationships between the continuous variables. Moreover, the correlation network analysis was displayed using the bootnet package of R software. The non-adjusted and adjusted differences in mean TIR % and HbA1c according to SES categories were tested using covariate-adjusted analysis of covariance (ANCOVA) models; in adjusted models, differences were controlled for sex, age, diabetes duration, HbA1c at baseline (before using FGM), insulin dose, BMI, time using insulin pump, and smoking behavior.

Repeated ANOVA models were used to test the mean change in HbA1c from baseline (before using the FGM sensor) to the end of follow-up according to deprivation index and mean net income categories, controlling for covariates. Interaction terms between HbA1c and SES categories were also tested. We also tested whether differences in HbA1c according to SES categories persisted at the end of follow-up using ANCOVA models controlling for sex, age, diabetes duration, insulin dose, BMI, time using insulin pump, and smoking behavior.

Logistic regression models were estimated using cardiovascular event and optimal glycemic control as dependent variables, SES categories as independent variables, and sex, age, diabetes duration, HbA1c before FGM, insulin dose, BMI, use of insulin pump, and smoking habit as covariates. Likewise, logistics regression models were also used to estimate the association between SES categories (independent variable) and optimal glycemic control, retinopathy, and nephropathy (dependent variables) controlling for potential covariates.

Although this is an observational study based on real-world clinical data, we considered that it would not be possible to conduct our study unless we had a minimum sample size of 500 subjects meeting the inclusion criteria, as smaller studies tend to overestimate OR estimates in logistic regression models [21].

The statistical analysis was performed using R, version 4.0.3 [22] and STATA 17.0 BE-Basic Edition statistical software (Lakeway Drive, College Station, TX, USA). The statistical significance was set at *p* < 0.05.

## Results

### Glycemic control and complications in patients with type 1 diabetes are related to socioeconomic variables

The final sample consisted of 1060 patients (49.0% female) ranging in age from 18 to 89 years (mean age 47.7 ± 15.0 years). The mean age at disease onset was 26.3 (± 15.7) years, and the mean disease duration was 21.5 (± 13.3) years. Of the study sample, 95.4% of the patients were users of multiple doses of insulin in a bolus-basal strategy, and 4.9% were open-loop CSII users. HbA1c before FGM placement was 62 ± 16 mmol/mol (7.9 ± 1.4%).

HbA1c levels prior to sensor placement showed significant differences between patient’s SES categories. Mean HbA1c values according to SES quartiles were, 60 ± 14 mmol/mol (7.6 ± 1.3%) mmol/mol in the first quartile (representing the best economic situation), 62 ± 14 mmol/mol (7.8 ± 1.3%) in the second quartile, 64 ± 16 mmol/mol (8.0 ± 1.4%) in the third quartile, and 65 ± 17 mmol/mol (8.1 ± 1.5%) in the final quartile (representing the worst economic situation) (*p* < 0.001). No statistically significant differences were observed in treatment modality (insulin pump or multiple daily insulin injections), BMI, insulin dosage, disease duration, or age at diabetes onset (Table 1).

**Table 1:** Characteristics of the study population according to socioeconomic status (SES) quartiles

**Variable**	**Obs ***n= 1060*	***Q1***	**Q2**	**Q3**	**Q4**	***p* value**
**Age**	47.7 (±15.0)	47.5 ±16.3	47.6 ±14.6	48.3 ± 14.3	46.6 ± 14.7	0.584^1^
**Sex women**	519 (49.0)	146 (50.0)	148 (51.0)	134 (46.5)	140 (48.3)	0.714^2^
**Age debut (years)**	26.3 (±15.7)	25.4 ± 17.6	26.8 ± 15.5	26.4 ± 14.8	25.3 ± 14.8	0.548^1^
**Net income/person/year (€)**	16985.2 ±6013.7	23671 ± 5745	18017±4334	14133 ± 2600	11852 ± 1806	<0.001^1^
**Multiple daily injections**	1011 (95.4)	275 (95.8)	275 (95.8)	262 (94.2)	271 (94.1)	0.651^2^
**Open loop Insulin pump (CSII)**	49 (4.6)	12 (4.2)	12 (4.2)	16 (5.8)	17 (5.9)	0.651^2^
**BMI (Kg/m**^**2**^**)**	25.8 (± 5.6)	25.6 ± 8.1	25.9 ± 4.5	25.6 ± 8.1	25.8 ± 4.3	0.279^1^
**Smokers**	215 (20.3)	45 (15.6)	51 (17.7)	65 (23.1)	68 (23.5)	0.040^2^
**Duration of diabetes (years)**	21.5 (± 13.3)	21.7 ± 14.9	20.5 ± 12.2	21.9 ± 12.8	21.1 ± 12.7	0.544^1^
**Mean pre-FGM HbA1c ( mmol/mol)**	7.9 ± 1.4 (62±16)	7.6 ± 1.3	7.8 ±1.3	8.0±1.4	8.1 ± 1.5	<0.001^1^
**Insulin (UDS/Kg)**	0.60 ± 0.24	0.57 ± 0.23	0.61 ± 0.25	0.63 ± 0.24	0.60 ± 0.24	0.126^1^
**Retinopathy**	269 (25.4)	70 (24.6)	69 (24.1)	79 (28.2)	67 (23.3)	0.542^2^
**Nefropathyª**	127 (12.7)	28 (11.1)	38 (14.5)	30 (11.5)	32 (11.8)	0.614^2^
**Stroke/Ishcemic cardiopathy**	55 (5.2)	7 (2.4)	16 (5.5)	17 (5.9)	20 (6.9)	0.081^2^

Table 1 describes the characteristics of the study sample

Differences were analyzed with the ANOVA^1^ and Chi-square test^2^ test

*Abbreviations: BMI* Body mass index

Regarding chronic complications of diabetes, a trend towards a higher prevalence of non-fatal cardiovascular events (ischemic heart disease and ischemic stroke) was observed as the SES decreased (*p* = 0.08). The prevalence of cardiovascular events was lower among individuals in the highest SES quartile compared to those in the lowest quartile (2.4% vs 6.9%, *p* = 0.010). No statistically significant differences were observed in complications such as nephropathy (*p* = 0.614) or retinopathy (*p* = 0.542) (Table 1).

**Fig. 1:**
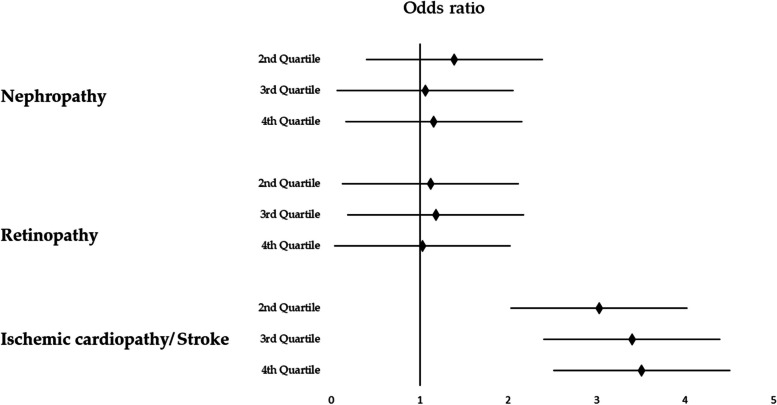
Risk of chronic complications of type 1 diabetes mellitus according to socioeconomic status (SES) quartiles All analyses used the first quartile of socioeconomic status (SES) as reference. The multivariate analysis for non-fatal cardiovascular events showed an odds ratio of 3.31 (*p* = 0.009) in the second quartile, 3.34 (*p* = 0.007) in the third quartile, and 3.80 (*p* = 0.003) in the last quartile of SES compared to the first quartile. No differences were observed among the different SES quartiles in relation to the prevalence of nephropathy or diabetic retinopathy. The analyses were adjusted for sex, age, diabetes duration, insulin dose, body mass index, insulin pump use, and smoking

Figure 1 depicts the risk of chronic complications according to SES categories determined by multivariate regression analyses controlling for sex, age, diabetes duration, insulin dose, BMI, insulin pump use, and smoking habit as covariates. The deprivation index was not a significant predictor of either retinopathy or nephropathy, but it was a predictor of non-fatal cardiovascular events; specifically, the risk of cardiovascular events was 3 times higher for those in the medium–low, medium–high, and higher deprivation categories as compared with those in the lower SES category.

Age (OR 1.07, 95% CI [1.04–1.10], *p* < 0.001), male sex (OR 1.91, 95% CI [1.13–3.22], *p* = 0.015), and disease duration (OR 1.03, 95% CI [1.02–1.05], *p* < 0.001) were also identified as risk factors for the presence of a non-fatal cardiovascular event. The remaining covariates did not show a statistically significant relationship with the presence of cardiovascular events. Descriptive data about retinopathy, and nephropathy, are presented in the Additional file 1: Fig. S2 and S3.

### Flash glucose monitoring (FGM) implementation improves HbA1c in all socioeconomic groups, without eliminating differences in time in range (TIR) and time above range (TAR) between the groups

**Fig. 2:**
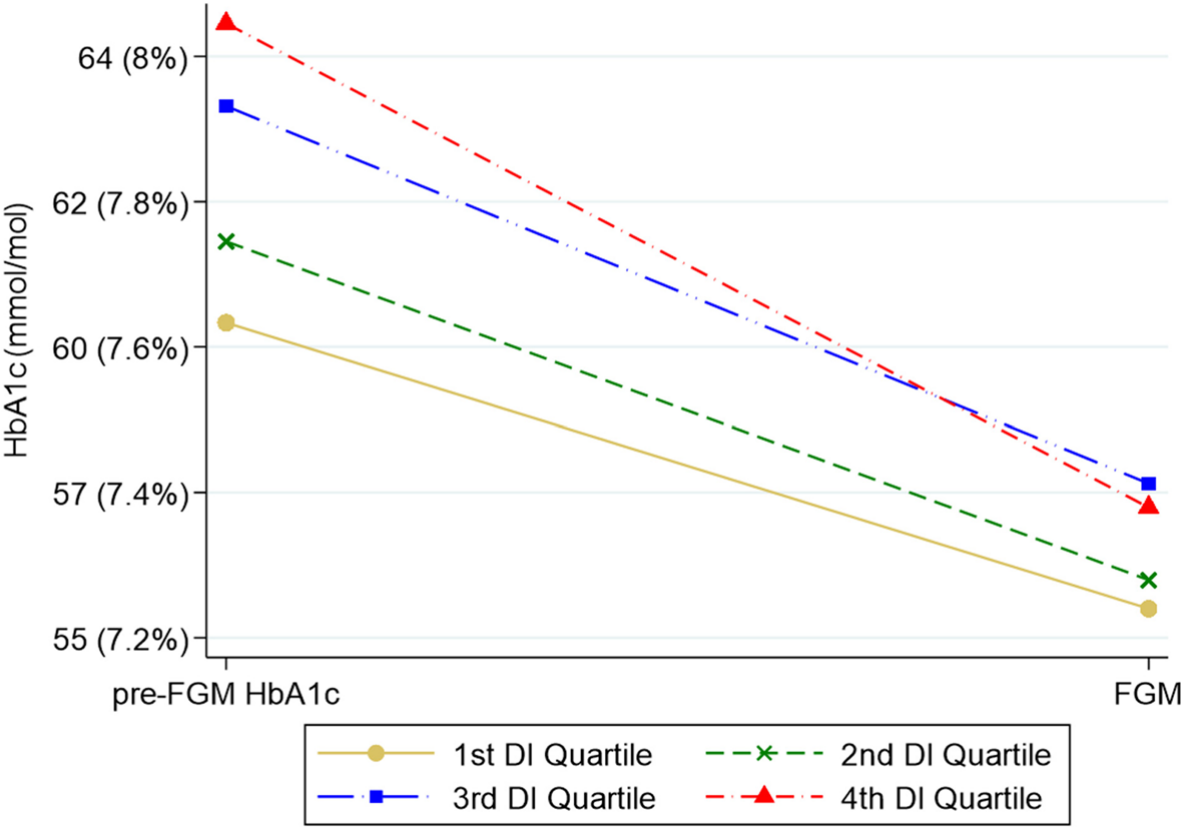
Change of mean HbA1c between baseline and end of 2-year follow-up with FGM DI: deprivation index. ªp value for Pillais’s trace ANOVA test for repeated measures with HbA1c at baseline and at the end of follow-up as intra-subject factors by SES quartile, controlling for time course of T1D and smoking behavior. Interaction term HbA1c* DI quartile (*p* = 0.021). The HbA1c of all groups of individuals improved (*p* < 0.001) regardless of their level of deprivation

After implementing FGM systems, a significant decrease in HbA1c was observed across all SES categories, ranging from 5 mmol/mol (0.4%) in the two highest SES quartiles to 7 mmol/mol (0.7%) and 6 mmol/mol (0.5%) in the two lowest quartiles (Pillai’s trace 0.069; *F* = 78.386; *p* < 0.001). An ANCOVA model controlling for covariates (sex, age, diabetes duration, insulin dose, BMI, time using insulin pump, and smoking behavior), showed that statistically significant differences (*p* = 0.001) in HbA1c levels persisted across all SES quartiles at the end of the follow-up period (Fig. 2).

After FGM start, SES was found to be significantly associated with the probability of optimal glycemic control (> 70% TIR with < 4% TBR) (*p* < 0.001) (Additional file 1: Fig. S4). The frequency of optimal control was higher in the highest SES quartile than in the lowest (27.8% vs 12.6%, *p* < 0.001) (Additional file 1: Fig. S5). Different FGM variables including TIR, TAR, and TBR improved as SES categories improved (Table 2). TIR was 7.1% higher in the highest SES group compared to the lowest (*p* < 0.001). Moreover, TAR > 180 mg/dL in the lowest SES quartile was 7% higher than that in the highest SES quartile (*p* < 0.001). Additionally, TAR > 250 mg/dL also exhibited a 3.4% difference among SES quartiles (*p* < 0.001). However, no statistically significant differences were observed in TBR < 70 mg/dL (*p* = 0.922) and coefficient of variation (*p* = 0.235) (Table 2).

**Table 2:** Glucose control parameters according to socioeconomic status (SES) quartiles

**Variable**	*Obs*	***Q1***	**Q2**	**Q3**	**Q4**	***p* value**
TIR	60.9 ± 17.7	64.9 ± 17.8	62.0 ± 17.1	60.3 ± 17.7	57.8 ± 17.4	<0.001
TBR<70 mg/dL	4.6 ± 4.8	4.8 ± 5.5	4.4 ± 4.2	4.6 ± 4.71	4.5 ± 5.0	0.922
TAR>180 mg/dL	34.5 ± 18.8	30.7 ± 18.7	33.8 ± 17.8	34.6 ± 18.9	37.7 ± 18.8	<0.001
TAR >250 mg/dL	11.7 ± 13.4	9.8 ± 12.8	10.9 ± 11.7	11.9 ± 14.0	13.2 ±13.6	<0.001
Coefficient of Variation	36.7 ± 7.1	36.1 ± 7.8	36.6 ± 6.5	36.8 ± 7.0	37.0 ± 7.2	0.235
Optimal control (%)	20.6 ± 40.0	27.9 ± 44.9	21.7 ± 41.3	20.2 ± 40.3	12.6 ± 33.3	<0.001

Optimal control is a composite variable composed of the combination of TIR > 70% and TBR < 4%

A consistent relationship is observed between higher socioeconomic status (SES) and better glycemic control as measured by glycosylated hemoglobin (HbA1c), time in range (TIR), and the percentage of individuals within optimal control. Statistically significant differences are observed in all variables (p<0.001). As socioeconomic status (SES) decreases, there is an increased duration of time spent above the thresholds of 180 mg/dL and 250 mg/dL. However, no differences are observed in the time below range (TBR) of 70 mg/dL or in the coefficient of variation

*TIR* Time in range (70-180mg/dL), *HbA1c* glycated haemoglobin, *TBR* Time below range(<70mg/dL), *TAR* Time above range (>180 mg/dL)

Finally, small differences were observed in the time as a Flash Glucose Monitoring (FGM) user between the quartile with the highest SES 2.0 (± 1.2) years and the quartile with the lowest SES 2.37 (± 1.6) years (*p* = 0.005). However, as we will see below, no independent association was observed between the time as a sensor user and glycemic control in the multivariate analysis. Therefore, the time as an FGM user does not seem to have a medium-term impact on glycemic control.

### SES is an independent risk factor of glycemic control in T1D patients using FGM

**Fig. 3:**
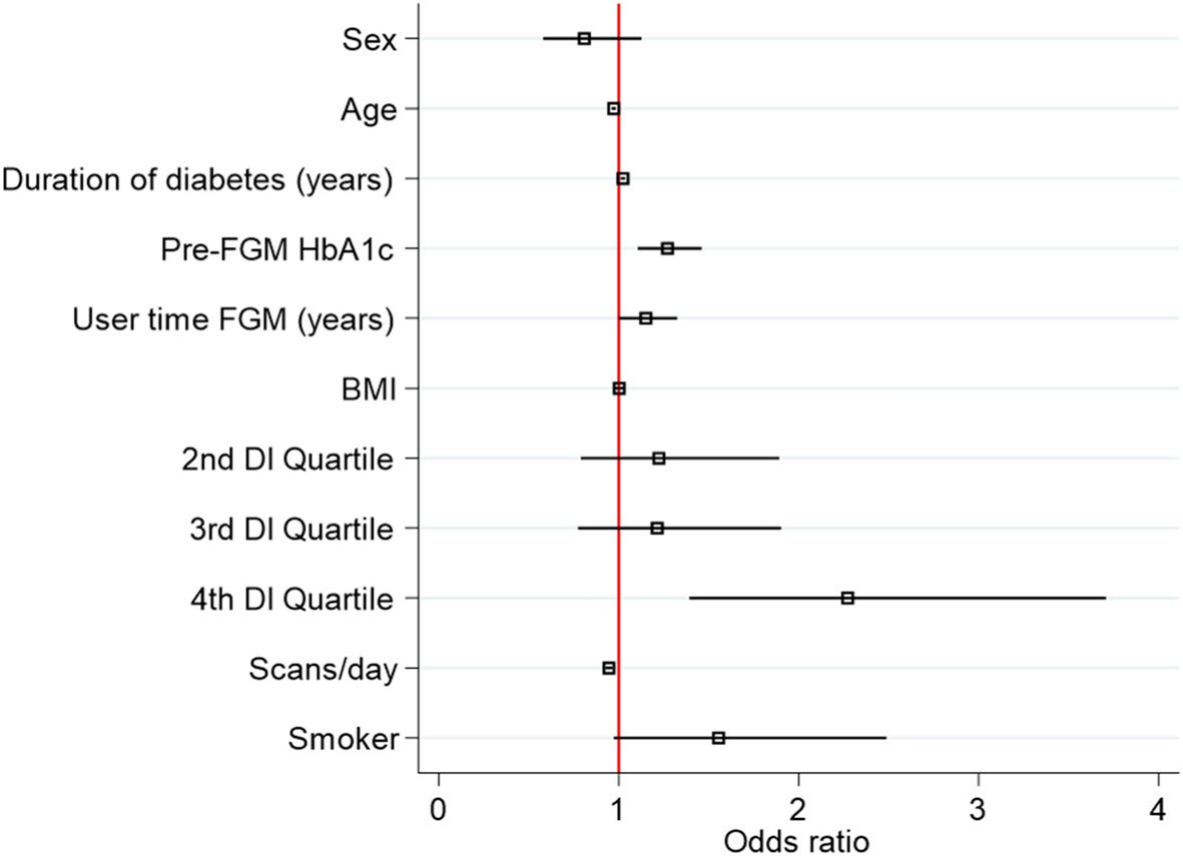
Multivariate logistic regression for chronic poor glycaemic control adjusted for covariates BMI, body mass index; DI, deprivation index; FGM, flash glucose monitoring; HbA1c, glycated hemoglobin. The only statistically significant variable not represented in the graph is the insulin dosage in units per kilogram (OR 13.99, *p* < 0.001). The fourth quartile of socioeconomic status (4th DI quartile) was found to be one of the most independently associated factors with being outside of optimal control (OR 2.27, *p* < 0.001)

In the logistic regression analysis, the lowest SES quartile was identified as a significant and independent predictor (*p* < 0.001) of not achieving optimal glycemic control compared to the highest quartile. Diabetes duration (years) (OR = 1.02; [95% CI: 1.01–1.03] *p* = 0.001), HbA1c at baseline (OR 1.27; [95% CI: 1.10–1.46] *p* = 0.001), and insulin requirements (units/kg body weight) (OR = 13.99; 95% [CI: 5.63–34.77] p < 0.001) were also factors favoring poor control. On the other hand, age (OR = 0.97; 95% [CI: 0.96–0.98] *p* < 0.001) and number of daily readings (OR = 0.94; 95% [CI: 0.92–0.96] *p* < 0.001) were protective factors for poor chronic control. Sex, BMI, use of an insulin pump, smoking, and time as an FGM user showed no association with optimal glycemic control (*p* > 0.05) (Fig. 3).

**Fig. 4:**
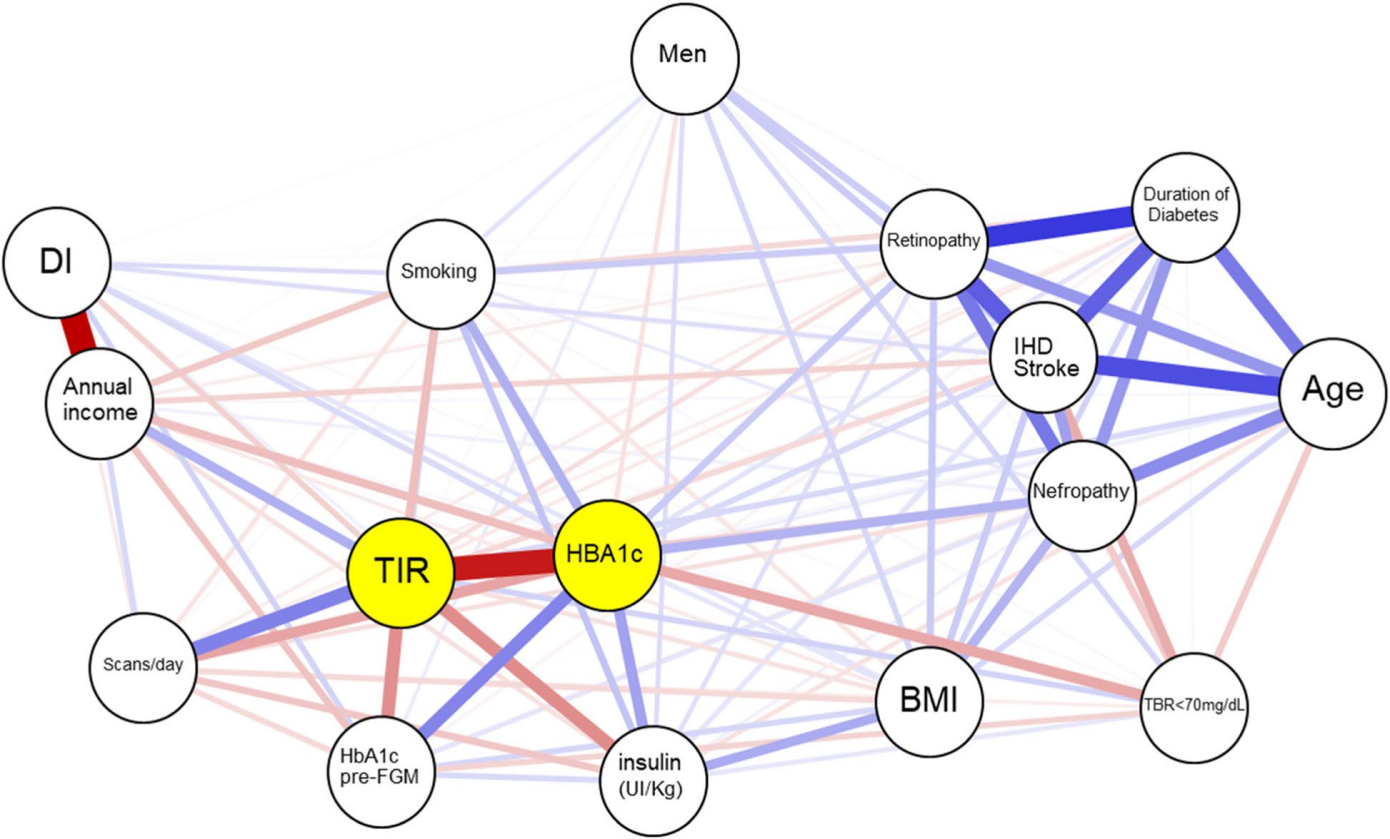
Network correlation plot between socioeconomic, glycaemic control, diabetes chronic complications, and potential confounding variables Only significant (*p* < 0.05) relationships are represented. The intensity of the blue color corresponds to the strength of positive correlations, while the intensity of the red color indicates the strength of negative correlations. BMI, body mass index; DI, deprivation Index; TIR, percentage of time in the range of 70–180 mg/dL; TBR < 70 mg/dL, percentage of time below 70 mg/dL; IHD, ischemic heart disease. This network plot illustrates the moderate association between socioeconomic parameters and glycemic control variables such as HbA1c and TIR, highlighting their association with common complications. Diabetic retinopathy, nephropathy, and cardiovascular events exhibit a strong interrelation, correlated with disease duration and age. Active smoking is similarly associated with parameters indicating poor glycemic control (lower TIR and higher HbA1c), as well as complications like diabetic retinopathy. Daily scanning is positively associated with improved glycemic control but not with chronic complications

Our models adjusted for covariates demonstrate that SES, assessed both as an index of deprivation and as average annual net income, not only represents a significant independent factor influencing chronic control, as mentioned before, but also correlates with macrovascular complications in T1D individuals using FGM. This finding is consistent with several other variables commonly employed in clinical practice, such as BMI, smoking habit, and diabetes duration. Figure 4 displays the relationship between various clinical and socioeconomic variables, influencing glycemic control, and in turn, chronic complications of T1D. In Additional file 1: Fig. S6 we show Pearson correlation coefficients between SES, glycemic control, and clinical variables.

## Discussion

We studied the influence of SES on glycemic control in individuals with free access to FGM systems used in accordance with guideline recommendations [22]. Our sample of patients with T1D met the standards of care, as they usually attended their appointments, had free access to technological systems, and used them as recommended by guidelines, as corroborated by the fact that most patients had HbA1c levels below 64 mmol/mol (8.0%) before sensor placement and 59 mmol/mol (7.5%) after sensor placement. We found that SES was a significant predictor of glycemic control and diabetes-related clinical outcomes, with a similar performance or even surpassing that of most clinical variables commonly employed in the management of T1D.

Although an association between T2D development and SES [23, 24] has been previously reported, this relation has not been observed for T1D [25], probably due to its different pathophysiology. However, in patients with T1D maintaining a good chronic control [6, 26, 27], the risk of complications [8, 9, 28], and even mortality [10, 11] are associated with the SES of individuals. The reasons behind these disparities in glycemic control in people with T1D showing different SES remain to be determined. Several potential factors could be responsible for these differences, including diet such as access to fresh fruit/vegetables versus fast food [28], physical exercise such as the availability of or access to spaces for physical exercise or the practice per se, health literacy [29], or regular visits to the endocrinologist [30].

The effect of socioeconomic deprivation on complications has been studied in both T2D [31] and T1D [8, 27, 32]. Our data support that the prevalence of ischemic heart disease and ischemic stroke is higher in the population with lower SES. Moreover, according to previous studies [10, 33], this association between macroangiopathic cardiovascular events and SES persisted after controlling for potential confounders. Regarding microvascular complications, although previous evidence supports that both the risk of diabetic nephropathy and its treatment are negatively influenced by more socioeconomically deprived environments [34–36], our findings did not substantiate the association between SES and the risk of nephropathy and retinopathy. A possible explanation, as some prior studies have suggested, is that new technologies can improve glycemic control and mitigate the risk of diabetes-related microvascular chronic complications regardless of each individual’s SES [36]. Thus, fairly good chronic control of most patients could help mitigate the association between the risk of microvascular complications and SES. One of the findings in our study relates to the association of age and better long-term glycemic control, while diabetes duration is correlated with poorer outcomes, with both variables showing a positive association. From our perspective, the impact of these glycemic variables is influenced by the age of onset of diabetes. Latent autoimmune diabetes in adults (LADA) is characterized by the onset of T1D in adulthood, as opposed to childhood or adolescence. LADA patients tend to have greater pancreatic reserve and lower insulin requirements compared to those who develop T1D at an earlier age [37, 38]. Consequently, individuals with LADA often maintain better glycemic control over time and may have a lower rate of diabetic complications compared to those who developed the condition in childhood.

Self-management technologies are essential to achieve optimal glycemic control and avoid complications of T1D. Likewise, although the advantages and free and open access to these technologies would suggest a reduction in socioeconomic disparities in health, it remains unclear whether these inequities can ameliorate with free access, as recent studies have reported the persistence of disparities in patients with lower SES because they had a lower use of technological devices [39, 40]. In this sense, the objective of this study was to determine whether SES, measured through a deprivation index by census tract, was associated with glycemic control in individuals with T1D who use FGM and feedback systems in a setting where these systems are covered by the national health system, readily available and used in accordance with guideline recommendations [41]. We found a significant improvement in HbA1c values across all groups following the placement of FGM sensors, with reductions ranging from 4 to 7 mmol/mol after more than 2 years of sensor use.

Our data showed that individuals in the highest quartile of socioeconomic deprivation were 50% less likely to achieve optimal T1D control than their counterparts in the least deprived quartile. Furthermore, this level of control was found to be independent of other influential factors in chronic diabetes management, such as sensor readings, pre-sensor HbA1c levels, duration of diabetes, age, as well as other variables including BMI, gender, and length of time as a FGM user. However, because patients with lower SES showed a greater improvement, the disparity in chronic control between SES categories decreased after CGM use. These findings suggest that the implementation of FGM technology among individuals with T1D, when used appropriately, can help narrow the gap in glycemic control observed between different SES levels.

This study has some limitations. The observational design of our study prevents us from guaranteeing the adequate control of potential confounders; however, because of the longitudinal nature of the observations, our estimates of the association between SES and glycemic control could be considered free from temporal ambiguity. Another limitation is that the deprivation index is not a variable that reflects individual SES but that of the group of people living in the same census tract*.* Theoretically, therefore, the analysis in this study should have used hierarchical models that take into account individual variables and census cluster variables, but there are multiple studies that support [42] the consideration of census cluster socioeconomic variables as individual ones.

 Moreover, another limitation that warrants acknowledgment is the insufficient documentation in the clinical records of lifestyle-related factors, such as diet, physical activity, substance abuse, the presence of other cardiovascular risk factors, or family history of vascular pathology. The lack of these data limited our capacity to explore the relationships between SES, long-term glycemic control, and the development of complications in type 1 diabetes T1D.

Finally, the short duration of follow-up did not allow us to assess whether the impact on mortality of the use of these glycemic control technologies is associated with SES, and we must assume this weakness of the analysis. Therefore, prospective studies are needed to better assess whether the effect of these technologies on mortality in people with T1D is independent of SES, which may not be generalized worldwide.

However, this study has some strengths. Our data were collected from three hospital-based areas thus the representativeness of this multisite sample is greater in terms of deprivation than if it only came from a single area. Moreover, since all the individuals in the sample were managed in the public health system, the homogeneity in the conditions of access to the CGM systems and the uniform criteria in the collection of data and biochemical determinations were guaranteed.

## Conclusions

In conclusion, SES exerts a significant influence on glycemic control and the risk of complications in individuals with T1D. Despite the substantial benefits of providing free access to technology and promoting its appropriate use, inequalities can ameliorate, but they persist. As previously suggested [43], it is essential to prioritize the identification and understanding of social determinants in diabetes to mitigate their negative impact on disease management. Addressing the causes of persistent disparities in outcomes and exploring effective strategies to bridge the remaining gaps are of paramount importance. This knowledge will enable personalized therapeutic approaches for individuals with diabetes. Addressing the causes of persistent disparities in outcomes and exploring effective strategies to bridge the remaining gaps are of paramount importance. This knowledge will enable personalized therapeutic approaches for individuals with diabetes.

### Supplementary Information

**Additional file 1:** **Fig S1.** Correlation between Net Income per Person and Deprivation Index: **Fig S2.** Diabetic retinopathy: description of the sample. **Fig S3.** Diabetic nefropathy: description of the sample **Fig S4.** Optimal control in Venn Diagram **Fig S5.** Optimal control (%) by SES quartile. **Fig S6**. Pearson correlation coefficients among socio-economic status, glycaemic control and clinical variables.

## Data Availability

The datasets used and/or analyzed during the current study are available from the corresponding author on reasonable request.

## Abbreviations

ANCOVA: Covariate-adjusted analysis of covariance
BMI: Body mass index
CGM: Continuous glucose monitoring
CSII: Continuous subcutaneous insulin infusion
CV: Coefficient of variation
FGM: Flash glucose monitoring
HbA1c: Glycated hemoglobin
OR: Odds ratio
SD: Standard deviation
SES: Socioeconomic status
T1D: Type 1 diabetes
T2D: Type 2 diabetes
TAR: Time above range
TBR: Time below range < 70 mg/dL
TIR: Time in range 70–180 mg/dL
