# Supplementary material for: Impact of socioeconomic status on chronic control and complications of type 1 diabetes mellitus in users of glucose flash systems: a follow-up study
Source: BMC Med. 2024 Jan 25;22:37. doi: 10.1186/s12916-024-03254-w (PMC10809494; doi:10.1186/s12916-024-03254-w)
Supplement: Supplementary file 1 — Additional file 1: Fig S1. Correlation between Net Income per Person and Deprivation Index: Fig S2. Diabetic retinopathy: description of the sample. Fig S3. Diabetic nefropathy: description of the sample Fig S4. Optimal control in Venn Diagram Fig S5. Optimal control (%) by SES quartile. Fig S6. Pearson correlation coefficients among socio-economic status, glycaemic control and clinical variables. [file 12916_2024_3254_MOESM1_ESM.docx]

**SUPPLEMENTARY MATERIAL S1** Correlation between Net Income per Person and Deprivation Index:


The image demonstrates a strong association between the deprivation index and the average net income per person in our sample (Pearson -0.750, p < 0.001)

**SUPPLEMENTARY MATERIAL S2**Diabetic retinopathy: description of the sample

| **Variable** | **Obs**  *n= 1060* |
| --- | --- |
| Retinopathy | 268 (25.4) |
| Grade 1 | 112 (10.6) |
| Grade 2 | 55 (5.2) |
| Grade 3 | 12 (1.0) |
| Proliferative | 89 (8.4) |

**SUPPLEMENTARY MATERIAL S3**

Diabetic nefropathy: description of the sample

| **Variable** | **Obs**  *n= 989* | **Definition** |
| --- | --- | --- |
| Nephropathy | 126 (12.7) | Grade >1 |
| Grade 1 | 865 (87.3) | GFR> 60 ml/min + MAU <30 mg/g |
| Grade 2 | 80 (8.1) | GFR>60 ml/min + MAU 30-30mg/g  GFR 45-59 ml/min + MAU <30 mg/g |
| Grade 3 | 18 (1.8) | GFR>60 ml/min + MAU >300mg/g  GFR 45-59 ml/min + MAU 30-300 mg/g  GFR 30-44 ml/min + MAU <30 mg/g |
| Grade 4 | 18 (1.8) | GFR 45-59 ml/min + MAU >300 mg/g  GFR 30-44 ml/min + MAU >30 mg/g  GFR 15-29 ml/min + MAU <300 mg/g |
| Grade 5 | 10 (1.0) | GFR 15-29 ml/min + MAU >300 mg/g GFR < 15 ml/min |

GFR: renal glomerular filtration rate
MAU: microalbuminuria

**SUPPLEMENTARY MATERIAL S4**Optimal control


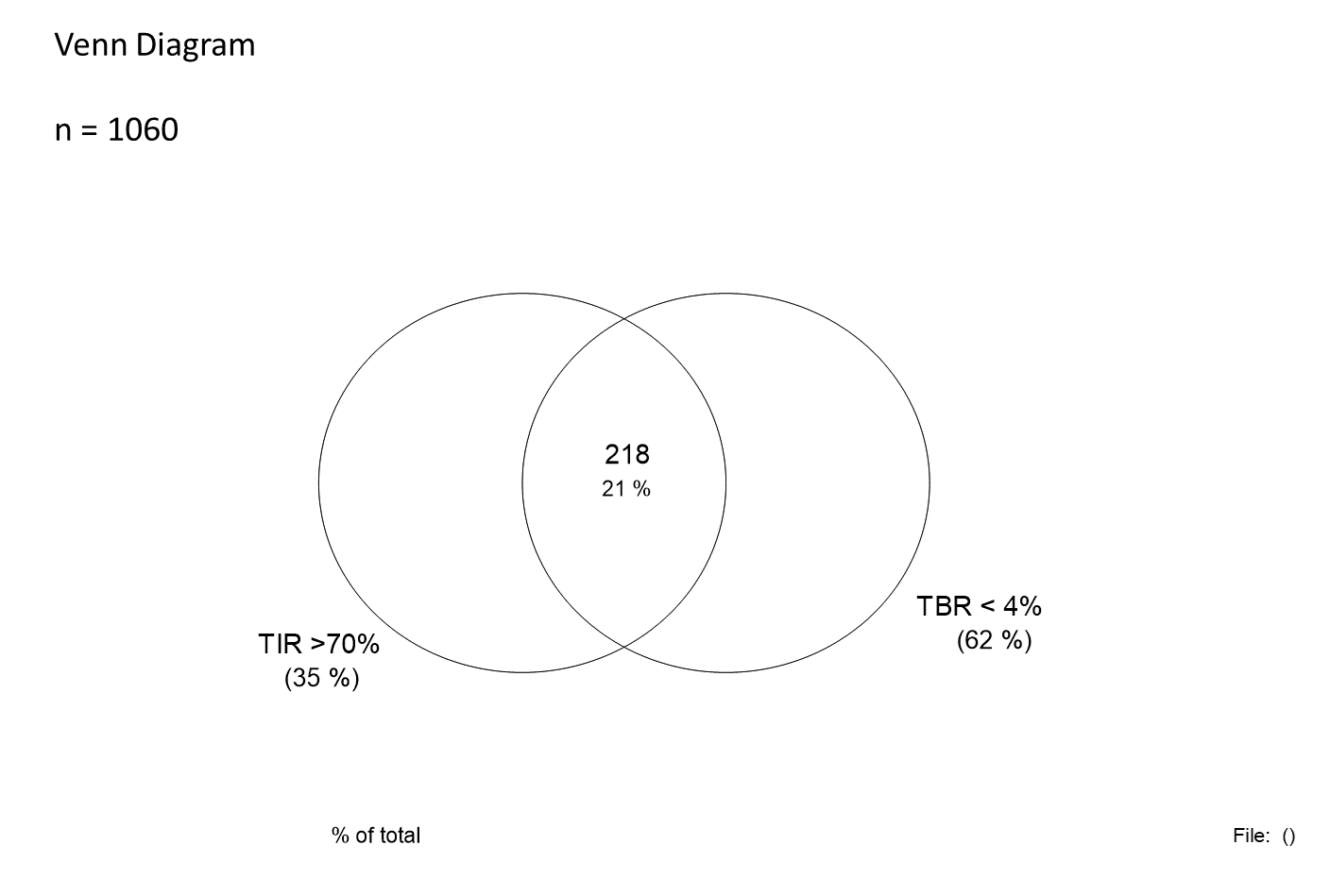


Optimal control is a composite variable composed of the combination time in range (70-180mg/dL) > 70% (TIR) and time below range (<70 mg/dL) < 4% (TBR).
The graph demonstrates that 35% of the sample has a time in range (TIR) exceeding 70%, and 62% of the sample has a time below range (TBR) of less than 4%. However, only 218 individuals out of 1060 (21%) meet both conditions simultaneously.

**SUPPLEMENTARY MATERIAL S5** Optimal control (%) by SES quartile.


Optimal control is a composite variable composed of the combination time in range (70-180mg/dL) > 70% (TIR) and time below range (<70 mg/dL) < 4% (TBR).The frequency of optimal control was higher in the highest SES quartile than in in the lowest (27.8% vs 12.6%, p < 0.001)

**SUPPLEMENTARY MATERIAL S6** Pearson correlation coefficients among socio-economic status, glycaemic control and clinical variables.**
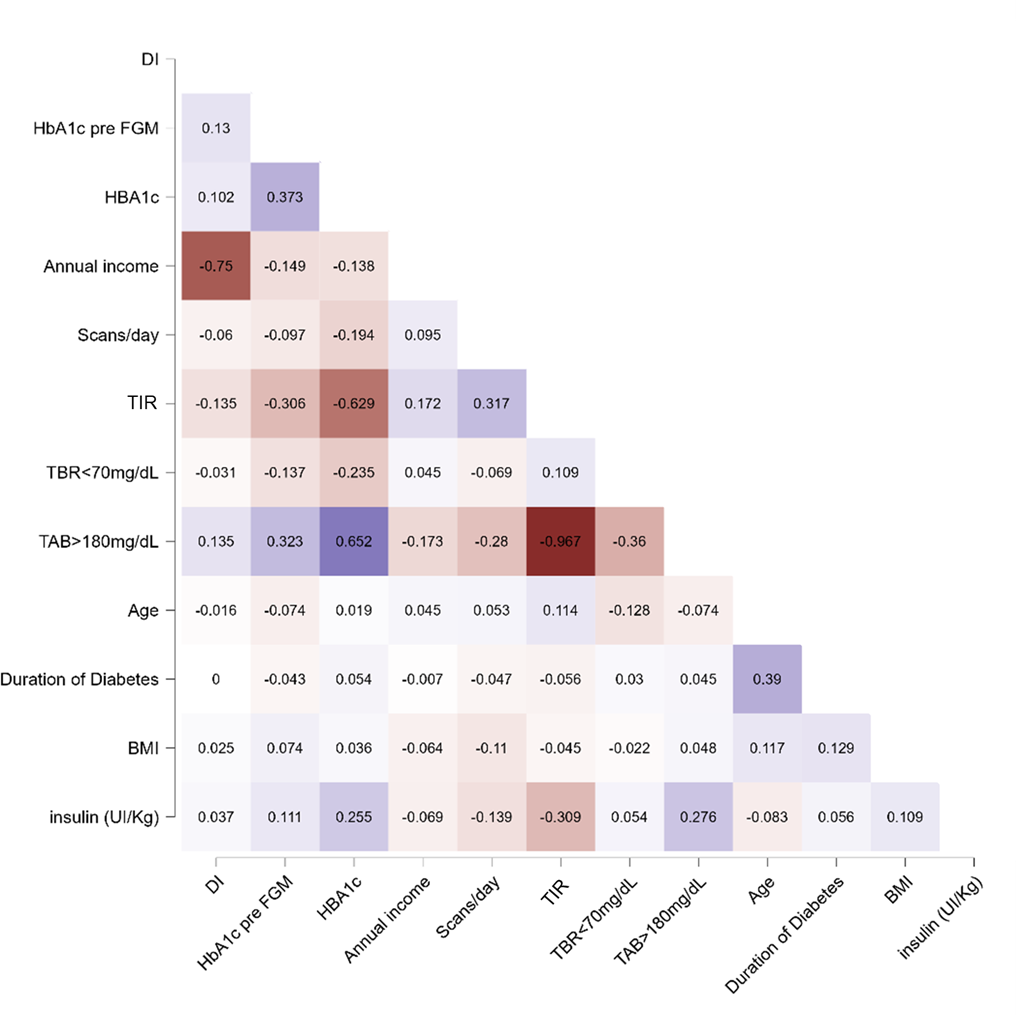
**

|  |
| --- |

DI: Deprivation Index; TIR: Percentage of time in the range of 70 – 180 mg/dL.; TBR<70mg/dL: Percentage of time below the range of 70mg/dL.BMI: Body mass index (kg/m^2^).
The intensity of the blue colour corresponds to the strength of positive correlations, while the intensity of the red colour indicates the strength of negative correlations.
A strong correlation is observed between glycosylated hemoglobin (HbA1c) and other glycemic parameters such as Time in Range (TIR), Time Below Range (TBR), and Time Above Range (TAR). Annual income shows a moderate association with TIR, indicating that higher income is associated with longer time within the target range, while it shows a negative association with TAR >180 mg/dL and HbA1c. Similar results are observed for the Diabetes Index (DI), with the exception that higher DI values are associated with increased poverty, leading to an inverse relationship with income. The number of scans is associated with lower HbA1c, TAR >180 mg/dL, and longer time in range. A higher insulin dose per kilogram is also postulated as a factor associated with poorer TIR, HbA1c, and TAR >180 mg/dL.
